# Microfluidic Technology for the Production of Well-Ordered Porous Polymer Scaffolds

**DOI:** 10.3390/polym12091863

**Published:** 2020-08-19

**Authors:** Pei Zhao, Jianchun Wang, Yan Li, Xueying Wang, Chengmin Chen, Guangxia Liu

**Affiliations:** 1 Energy Institute, Qilu University of Technology (Shandong Academy of Sciences), Jinan 250014, China; wangjc@sderi.cn (J.W.); liyan@sderi.cn (Y.L.); chencc@sderi.cn (C.C.); liugx@sderi.cn (G.L.); 2 School of Energy and Power Engineering, Qilu University of Technology (Shandong Academy of Sciences), Jinan 250353, China; 3 Key Laboratory of Interfacial Reaction & Sensing Analysis in Universities of Shandong, School of Chemistry and Chemical Engineering, University of Jinan, Jinan 250022, China; chm_wangxy@ujn.edu.cn

**Keywords:** well-ordered porous scaffolds, microfluidic technology, pore structure, tissue engineering

## Abstract

Advances in tissue engineering (TE) have revealed that porosity architectures, such as pore shape, pore size and pore interconnectivity are the key morphological properties of scaffolds. Well-ordered porous polymer scaffolds, which have uniform pore size, regular geometric shape, high porosity and good pore interconnectivity, facilitate the loading and distribution of active biomolecules, as well as cell adhesion, proliferation and migration. However, these are difficult to prepare by traditional methods and the existing well-ordered porous scaffold preparation methods require expensive experimental equipment or cumbersome preparation steps. Generally, droplet-based microfluidics, which generates and manipulates discrete droplets through immiscible multiphase flows inside microchannels, has emerged as a versatile tool for generation of well-ordered porous materials. This short review details this novel method and the latest developments in well-ordered porous scaffold preparation via microfluidic technology. The pore structure and properties of microfluidic scaffolds are discussed in depth, laying the foundation for further research and application in TE. Furthermore, we outline the bottlenecks and future developments in this particular field, and a brief outlook on the future development of microfluidic technique for scaffold fabrication is presented.

## 1. Introduction

In tissue engineering (TE), porous scaffolds serve as valuable three-dimensional (3D) supports for cell growth and the subsequent tissue formation [1,2]. The ideal scaffold needs to have a porous structure, good biocompatibility, degradability and certain biomechanical properties [3]. Among those, the pore structure (pore size, shape, porosity and pore interconnectivity) of the scaffolds is a key parameter that directly affects the secretion of the extracellular matrix, the degree of cell movement, cell differentiation and signal transduction [4,5]. Small pore size and broad pore size distributions can easily cause low cell seeding efficiency and heterogeneous distribution of cells [6]. Highly ordered, interconnected pores and uniform spatial structure of scaffolds are preferred in TE. The ordered structure of the scaffolds serves as the template and anchor for the protein array, and enables the cells to selectively adhere to a specific location in the scaffold [7]. The fully interconnected morphology of scaffolds permits proper exchange of liquids, migration of cells and vascularization [8,9]. Uniformity of pores allows individual cells grown within the scaffolds to experience a similar environment to proliferate and differentiate [10]. Also, the mechanical properties and the degradation rate in each region of the scaffold should be same to guarantee steady performance throughout the whole scaffold [11,12]. In addition, TE scaffolds are often modified or loaded with bioactive molecules, such as growth factors. In a uniform spatial structure, it is easy to obtain an ideal release behavior of bioactive molecules. Herein, the control of the fine structure is important in the preparation of porous scaffolds, because it directly impacts the scaffold’s mechanical stability and its regulation on cellular behaviors. That is of great significance to further elucidate the influence of scaffold structure on both intracellular and intercellular interactions.

To achieve a certain scaffold topology many solutions have been recently proposed in the field of material sciences, such as freeze drying [13,14], gas forming [15,16], solvent casting/particulate leaching [17,18,19], emulsion template [20,21] and electrospinning [22,23,24]. The advantages and limitations of these traditional methods are summarized in Table 1. 

Uniform pores and well-defined pore geometry, as well as interconnection between the pores, cannot be simultaneously and precisely generated by most of these methods. Tailoring the morphology of the macroporous structure remains one of the biggest challenges in the preparation process of scaffolds. In recent decades, numerous studies have sought to control pore size and size distribution to create well-ordered porous scaffolds by emerging approaches [25,26,27,28,29,30], and their advantages and limitations are summarized in Table 2. Among them, a templating method based on evaporative cooling, which was called breath figure, was applied to produce well-ordered porous films [26]. Nevertheless, the temperature and humidity must be strictly controlled and volatile solvents were used in the process of ordered porous structure formation. The materials used in this method were limited to amphiphilic copolymers, and the hydrophilic-hydrophobic balance of the copolymers was important to obtain well-ordered porous films. Lately, Xia et al. prepared inverse opal scaffolds by templating against cubic close packed lattices of monodispersed microspheres [31,32]. Inverse opal scaffolds had high porosity, pore size uniformity and good connectivity, but, multi-step operation and organic solvent removal were needed and solvent residue may be a biological toxicity affect the adhesion of cell growth. With the development of advanced micromachining technology, researchers have developed 3D printing technology to prepare highly ordered porous scaffolds [33,34,35,36,37]. This method was programmed to produce scaffolds that meet the needs of various defect shapes, but it required expensive machine control and time-consuming per-pixel writing. Meanwhile, the obtained scaffolds had poor penetrability and low resolution, and cannot reach the nanoscale ultrastructure. Moreover, the lack of suitable ink materials (especially high viscosity polymers) limited its application in mimicking the composition of human tissue. Therefore, available well-ordered porous scaffolds preparation technologies usually require multiple steps or harsh experimental conditions, and have limitations in biomedical and tissue engineering application. Hence, a controllable and practical method is urgently needed, that would expand biomedical applications of scaffolds and increase the utilization of a wide category of polymers, such as natural polymers.

In past decades, microfluidic technology has become a highly interdisciplinary science and technology to deal with single-phase or multiple-phase fluids in microchannels [38]. Droplet microfluidics has a wide range of applications in biochemical analysis and the generation of nanometer/microscale materials [7,39,40]. The monodispersity of generated materials from droplet microfluidics are much higher than those from conventional methods. Moreover, the structure and function of the materials can be adjusted flexibly [41,42]. Expect for obtaining polymer microspheres, microfluidic droplets were further utilized as template for the production of 2D and 3D porous scaffolds. The porous structure is highly ordered and overcome the structure defects of traditional scaffolds, such as wide pore size distribution and irregular geometries of pores [43,44,45]. In addition, this method is easily manipulated, low cost and do not require a complex setup, improving the reproducibility and simplifying the preparation process. Therefore, microfluidic technology offers an excellent platform for synthesizing high quality libraries of porous scaffolds [46].

**Figure 1:**
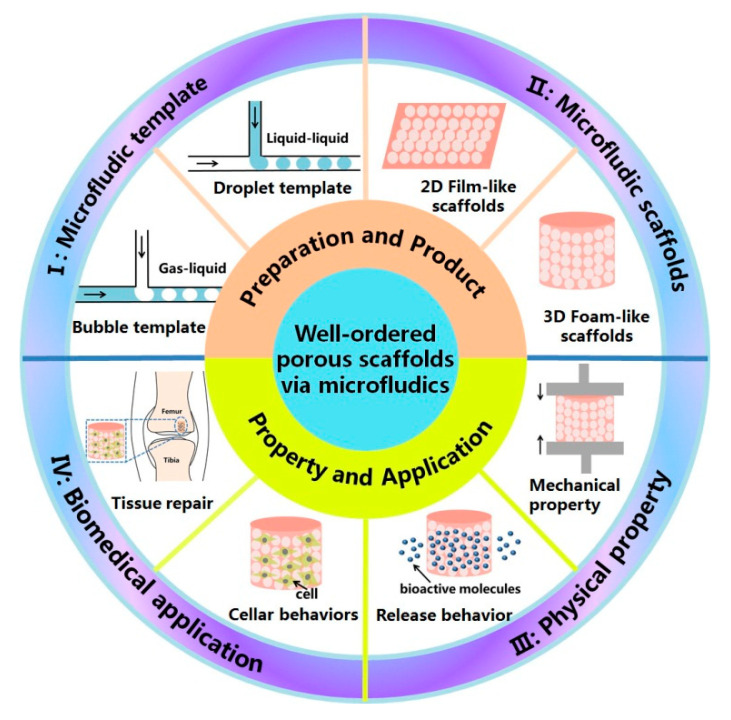
Schematic illustration of preparation and property of well-ordered porous scaffolds via microfluidic technology. Microfluidic template used for preparing the scaffolds, including bubble template and droplet template; The obtained microfluidic scaffolds, including 2D film-like scaffolds and 3D foam-like scaffolds; Physical property of the scaffolds, for example, mechanical property and release behavior of bioactive molecules; Biomedical application of the scaffolds, for example, in the field of tissue repair and cellar behaviors study.

To date, several remarkable reviews on the fabrication of polymeric scaffolds have already been published, but a comprehensive review about microfluidic fabrication of well-ordered porous scaffolds is still lacking. In this short review, we introduce the basic fabrication methods of porous scaffolds using microfluidic technology, the pore structure and biomedical applications of microfluidic scaffolds (Figure 1). Finally, the major challenges in this area and opinions on its future developments are proposed.

## 2. Well-Ordered Porous Scaffolds

**Figure 2:**
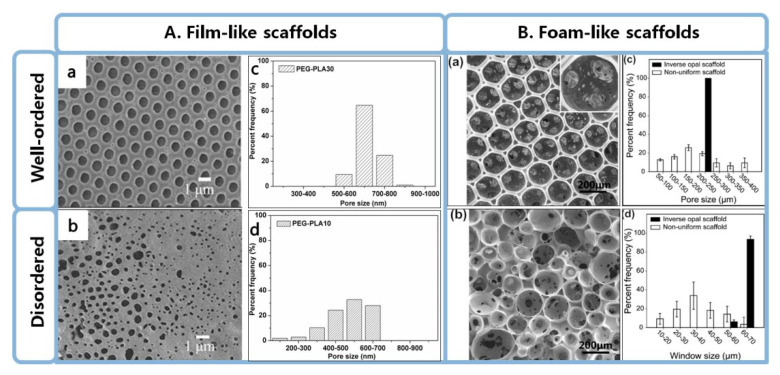
(**A**) SEM images and size distribution of (a,c) PEG-PLA30 and (b,d) PEG-PLA10 porous films fabricated by the breath figure templating technique. Percent frequency (%) refers to the percentage of the pore size within a certain range. Reprinted from [25] with permission. Copyright 2015 Elsevier. (**B**) SEM images of (a) an inverse opal scaffold and (b) a non-uniform scaffold, and size distribution of (c) pores and (d) windows (or the holes connecting adjacent pores) in each scaffold. The black and white bars correspond to the inverse opal and non-uniform scaffolds, respectively. Reprinted from [32] with permission. Copyright 2010 American Chemical Society.

As we all know, the structures and properties of the scaffolds are vital in determining the cellular response and fate in TE [5,47,48]. Well-ordered porous scaffolds refer to those have highly ordered microporous textures with regular pore shape, uniform pore size and ordered pore arrangement. Recent literature reports indicated that well-ordered porous structures performed better in the study of cellular behaviors than disordered porous scaffolds [25,27,32,49]. In this short review, two kinds of well-ordered porous scaffold are detailed introduced, including 2D film-like scaffolds and 3D foam-like scaffolds. 2D films usually have regular pore shape and narrow pore size ditribution, such as poly(ethyleneglycol)-block-poly(lactic acid) (PEG-PLA) honeycomb-like structure prepared by breath figure (Figure 2A) [25,26]. 3D foam scaffolds usually have uniform pore size with a long-range ordered and well controlled interconnectivity, such as inverse opal scaffolds by closely packed lattice of monodispersed microspheres (Figure 2B) [32]. The ordered pore structure of scaffolds has important effects on physical properties of scaffolds and cellar behaviors on scaffolds. Microfluidic technology that could be used to prepare well-ordered or tailor-made porous polymer scaffolds is explained in the following section. 

## 3. Well-Ordered Porous Scaffolds Based on Microfluidic Technology

**Figure 3:**
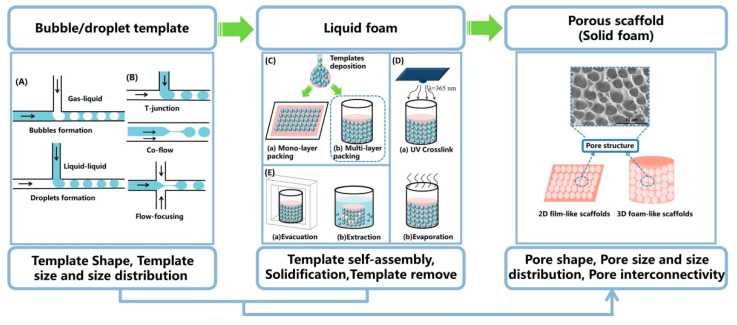
Schematic diagram: making porous scaffold with controlled structures via microfluidic technology. (**A**) Generation of templates, including bubbles formation and droplet formation; (**B**) Three common microfluidic channel geometries, including T-junction, co-flow and flow-focusing; (**C**) Self-assembly of templates, including mono-layer packing and multi-layer packing; (**D**) Solidification of external phase, including UV crosslink and evaporation; (**E**) Remove of templates, including evacuation and extraction.

As mentioned above, there are many types of ordered porous scaffolds, such as 2D films and 3D foams, as well as not mentioned fibers and microparticles [50]. This review mainly introduces microfluidics fabrication of 2D films and 3D foams, especially 3D foams. The foam-like scaffolds are actually solid foams with an open-pore structure, which are formed by solidification of liquid foams. Foaming templates and emulsion templates are usually used to prepare liquid foams, and the structure of template is one of the key factors for liquid foams (Figure 3). In recent years, the potential of microfluidic technology in generating a packed array of monodisperse gas-in-liquid bubble or liquid-in-liquid droplet was exploited [30,49,51]. Microfluidic bubbles and droplets act as sacrificial templates to prepare well-ordered and customizable shape porous scaffold [27,28,29,30].

The primary advantage of the microfluidic template is the monodispersity and also the generation of these homogenous templates is precisely and effectively controlled and tuned in a passive or active manner [52]. That is critically important to well-ordered porous scaffold preparation, especially for scaffolds with special structure requirements. Based on the flow behaviors, the general principles used in microfluidics for fabricating scaffolds are divided into gas-liquid interfaces segmented flow and liquid-liquid segmented flow, which respectively format bubble template and droplet template (Figure 3A). T-junction, co-flow and flow-focusing are three common microfluidic channel geometries, which are used and described to create monodisperse template (Figure 3B) [42,53]. In these channels, the continuous phase squeezes the dispersed phase leading to template formation [40]. Then, the template is collected inside a customized mold at the end of the tube and spontaneously self-assembled into crystal-like structures (Figure 3C) [54,55]. After external solidification and template removal, a scaffold with uniform porous texture is produced (Figure 3D,E) [38,45]. The pore size and interconnection of the scaffolds were tailored accurately by the template size, template volume fraction. The specific anatomical shape of the scaffolds was directly fabricated with a mold into square films or cylindrical foam. This template-assembly-based fabrication is easily reproduced and practical, and the final morphology of scaffolds fits the TE requirements.

### 3.1. Bubble Template

As reported, scaffolds have been prepared by solidifying liquid foams rapidly to form solid foams [56]. In a traditional gas-in-liquid foaming technique, a high-pressure hydrophobic gaseous phase (e.g., argon) is dispersed into a polymer solution inside a stirred reactor [57]. This method is an energy-saving and cell friendly approach, because the template does not need to be removed and toxic organic solvents are avoided in the preparation process. However, traditional foaming methods (e.g., mechanical whipping) cannot control the size and size distribution of the bubbles well, thus, resulting in polydisperse scaffolds. Microfluidic technology helps to overcome this lack of control over the bubble size and size distribution: with microfluidics, monodisperse bubble templates can be generated to form liquid foams, and the solidification of liquid foams leads to scaffolds with well-defined pore sizes and narrow pore-size distributions [58,59].

**Figure 4:**
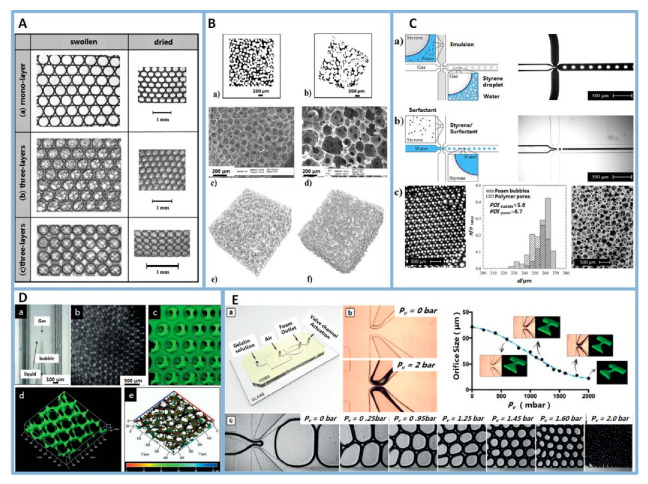
(**A**): Ordered polymerised foam layers in the swollen and dried state obtained by confining the initially liquid foam between narrowly spaced glass plates. (a) mono-layer packing, (b) three-layers packing in the (1 1 1) direction of the fcc packing, (c) three-layers packing in the (1 0 0) direction of the fcc packing. Reprinted from [60] with permission. Copyright 2009 Elsevier. (**B**): Microcomputed Tomography (μCT) slices of the PVA scaffolds produced with (a) the microfluidic foaming technique and (b) gas foaming technique, SEM of the (c) microfluidic foaming and (d) gas foaming PVA scaffolds, 3D rendering of the (e) microfluidic foaming and (f) gas foaming PVA scaffolds. Reprinted from [63] with permission. Copyright 2012 American Chemical Society. (**C**): Schematic drawing and photograph of the generation of a monodisperse foamed styrene-in-water emulsion (a) and of a monodisperse water-in-styrene emulsion (b). (c) Optical microscopy of emulsion, initial bubble size and final pore size distribution and SEM of monodisperse polystyrene foam. Reprinted from [69] with permission. Copyright 2015 Wiley. (**D**): Optical micrographs of (a) microfluidic device and (b) bubbles, (c–e) confocal microscopy of microfluidic scaffold. Reprinted from [64] with permission. Copyright 2014 Wiley. (**E**): (a) Schematic of the *vFF* chip, (b) optical micrographs of the device, (b) optical micrographs of the vFF during foaming for different pressure. Reprinted from [71] with permission. Copyright 2019 Wiley.

The first examples of highly structured porous polymer scaffolds synthesised from microfluidic bubbling were reported in 2009 [54,60]. Monodisperse microbubbles were obtained by a microfluidic method and self-assembled into crystalline foam structures spontaneously. In a balanced liquid foam (especially one composed of monodisperse small size bubbles), the capillary forces are strong enough to maintain a certain height region, so that the bubbles remained spherical and self-organized into crystalline structures. The structures were related to the number of bubble layers and the “crystal direction” of the “bubble crystals”. For instance, when the bubbles were in a monolayer packing, the bubbles were arranged into a classical hexagonal structure (Figure 4A(a)). When the bubbles were in three-layer packing, in the (1 1 1) direction of the face-centered-cubic (fcc) packing, the bubbles were arranged into a hexagonally close-packed structure at the surface (Figure 4A(b)), whereas, in the (1 0 0) direction of the fcc packing, a square arrangement of bubbles was observed at the surface (Figure 4A(c)). Later on, microfluidic bubbling was extensively adapted to more monomer and polymer solutions to prepare foams [61,62]. Examples of well-ordered porous foams are poly(vinyl alcohol) (PVA) foams (Figure 4B) [63], gelatin-based foams (Figure 4D,E) [64,65], alginate foams [66,67], as well as chitosan-based foams [49], polyacrylamide foams [60], polyurethane foams [68] and polystyrene foams (Figure 4C) [61,69]. Recently, researchers have developed tailor-made porous polymer scaffolds using microfluidics, such as monodisperse porous scaffolds, polydisperse porous scaffolds [49] and graded porous scaffolds [70,71]. For example, a valve-based flow-focusing (*vFF*) device was used to generate foams with controlled bubble size and the pore size of foams varied in the range of 80–800 μm (Figure 4E). In addition, the *vFF* device was combined with 3D printing technology to manufacture scaffolds with hierarchical pore size and porosity. The controlled changes of foam structure will allow the systematic study of structure-property relationships. Furthermore, a graded porous scaffold is a suitable candidate for applications in interfacial TE, such as bone TE.

The generation of liquid foams with controlled structures requires the manipulated generation of gas bubbles. The generation of gas bubble is related to the structure of the microfluidic device, the foam formulation, the viscosity of liquid phase, the liquid flow rate and the applied gas pressure. Common microfluidic device used for preparing gas bubbles are show in Figure 3B. For example, a flow focusing chip was used to prepare PVA foams and the channel parameters were as follows: the gas inlet was 300 μm, the liquid inlet was 300 μm, the orifice was 100 μm, the outlet channel was 700 μm and the depth of the chip was 150 μm. In fixed microfluidic chip and foam formulation, the diameter and shape of the bubble are mainly determined by the liquid flow rate (*Q*_l_) and the applied gas pressure (*P*_g_). For instance, in PVA foam production (Figure 4B), the system reached stability at *Q*_l_ = 2.25 µL/min and *P*_g_ = 67 kPa. The results showed that the diameter of the bubbles depended linearly on the gas pressure and inversely on the liquid flow rate [63]. Besides, the viscosity of the liquid phase (*η*) also influenced the bubble size. In alginate foam production, the accessible range of the bubble size tended to be narrow as the *η* increased. At the same *Q*_l_ = 40 µL/min, the range of bubble size was 150–300 μm in the case of 10% w/w alginate solution (*η* = 273 mPa·s), whereas, in the case of 15% w/w alginate solution (*η* = 1100 mPa·s), the range of bubble size was 150–200 μm [66,72]. Additionally, microfluidic technology was also used to prepare scaffolds with controlled polydispersity by using different microfluidic chips. For example, Andrieux et al. modified the microfluidic flow-focusing technique by gas pressure oscillates periodically and generated solid foams with controlled polydispersity [49].

Microfluidic bubble templating is a powerful tool to generate well-ordered scaffolds. However, one of its challenges is to control the pore interconnectivity, which strongly depends on the volume fraction of the gaseous phase and formulation of foams. In experience, foams with a gaseous fraction above to 80% tend to show a high degree of interconnection [27]. A high gaseous fraction, meaning high pressure value, is a great challenge to the sealing of microfluidic device. Besides, foams which is made up of different formulations form different pore structures: cross-linkable biopolymers, such as chitosan or alginate almost always produce open-cell foams, whereas synthetic polymers, such as polyurethane and polystyrene usually form either open- or to closed-hole foams depending on the choice of surfactant and the locus where the polymerization is initiated [61,68,69]. Therefore, by increasing the volume fraction of gas phase reasonably and choosing appropriate material formulation, the porous scaffold with good pore interconnectivity could be successfully prepared. Another challenge is liquid foams stability during the collecting and crosslinking step. In fact, there are the kinetics instabilities in liquid foam, such as coalescence and coarsening. The longer collecting time, the more pronounced effects arise in liquid foams [49,73,74]. Meanwhile, crystalline structures and the dispersity of liquid foams are also affected by the degree of crosslinking. The ordered honeycomb structure near the surfaces of the scaffolds and the broad size distribution of the pores in deep inside the scaffolds are produced by the non-uniform crosslinking [63,66]. One approach to this issue is the addition of solid particles or novel surfactant to stabilize the liquid foams, but, this method is rarely used for preparation of porous scaffolds. Another possibility to produce stable foams is to change the bubble template into another more stable template, such as droplet template. Due to the small density difference between the two phases, the aging effect of droplet template is less than that of the bubble template. Droplet template based on microfluidics is explained in the following section. 

### 3.2. Droplet Template

**Figure 5:**
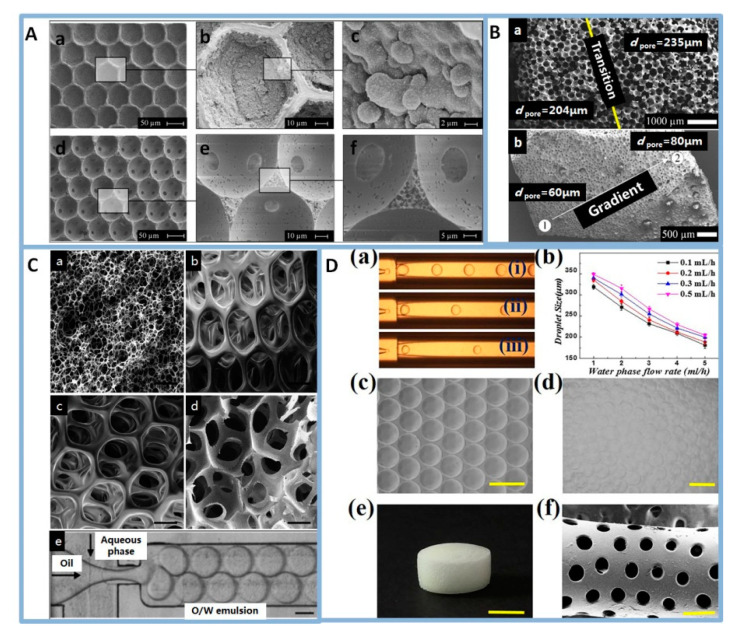
(**A**): SEM pictures of polymer foams with the locus of initiation being at the o/w interface using potassium persulfate (KPS) (a–c) or in the bulk phase using azobis-(isobutyronitrile) (AIBN) (d-e) at different magnifications. Reprinted from [82] with permission. Copyright 2017 American Chemical Society. (**B**): Polystyrene foams with a pore size and density gradient from foamed emulsion templating (a) and emulsion templating (b). Reprinted from [80] with permission. Copyright 2017 Wiley. (**C**): SEM micrographs of scaffolds fabricated through (a) conventional HIPE formation and (b–d) microfluidics. (e) Optical micrograph of the flow focusing chip, the scale bars measure respectively (a) 30 μm, (b–e) 100 μm. Reprinted from [83] with permission. Copyright 2014 Royal Society of Chemistry. (**D**): (a) Real-time image of the droplet template in the microfluidic device with different inner and outer flow rates, (b) the relationships of the pore size to the outer flow rates and the inner flow rates, (c,d) microscopic photographs of the monolayer droplet and multilayer droplets, (e) a general view of the porous scaffold, (f) the microstructure of the porous scaffold that fits the rat uterus, scale bars measure respectively (c,d,f) 500 μm and (e) 5 mm. Reprinted from [51] with permission. Copyright 2019 Elsevier.

The emulsion templating technique, which has flexibility in tailoring the structure of porous materials, is a fascinating technique for fabricating porous scaffolds [20,21]. High internal phase emulsions (HIPEs), containing over 74 vol% of the internal phase dispersed in the continuous phase, have been usually used for the preparation of polymer scaffolds. The polymerization of continuous phase results in what was known as polyHIPE. A porous polymeric material was acquired after solidification of the continuous phase and removal of the dispersed phase [75,76,77]. However, the average diameters of pores and interconnects were smaller than 100 and 40 μm, respectively, which impeded cell migration. Moreover, the pore size and the connecting pore size were typically highly dispersed [78]. Droplet microfluidics takes the full advantage of precisely handling liquids at the micrometer scale to produce uniform droplets with highly tunable size, structure and chemical composition. That was applied to fabricate polymeric particles [42], fibers [79] and foams [80]. Thanks to the flexibility of microfluidic technology, many porous biomaterials were conveniently fabricated, and their component, porosity, and shape were easily adjusted. The big advantage of the microfluidic emulsion technique is the fact that the droplet size and size distribution of the emulsion template are accurately controlled and tailored. Quell et al. first synthesized monodisperse w/o emulsion templated polymer foams based on microfluidic technology [69]. Later on, a large amount of monomer-based microfluidics emulsions and polymer-based microfluidics emulsions were developed to prepare well-ordered porous foams. In monomer-based systems, the solid foam is produced by the polymerization of monomers. A w/o emulsion, which styrene/divinylbenzene as hydrophobic monomer and water as the dispersed phase, is one of the most studied templates (Figure 5A,B) [61,81,82] but this material is not widely used in TE scaffolds due to its lack of biocompatibility. Highly ordered and precisely tailored porous dextran-methacrylate polyHIPE gel was also reported using microfluidics, this gel represented a new class of scaffolds for application in TE (Figure 5C) [83]. In polymer-based systems, the solid foam is prepared by cross-linking polymer melt or polymer liquid foam. Gelatin methacryloyl foam (Figure 5D) [51], poly(lactic-*co*-glycolic acid)-block-poly(ethylene glycol) (PLGA-*b*-PEG) foam [28], PVA foam [29,30,84], alginate foam [51] were prepared by microfluidic emulsion templating. These polymers are either artificial biocompatible materials or natural polysaccharide materials, which are suitable used as TE scaffold. More importantly, by adjusting the preparation parameters of microfluidic droplets, an emulsion template with gradient droplet size was generated, whose polymerization also lead to a polymer with a pore size gradient [80,85]. For example, a pressure-driven microfluidic device was introduced to produce polystyrene foams with a pore size gradient by foamed emulsion templating (FET) and emulsion templating (ET). The pore sizes of foam via FET are 204 μm and 235 μm, respectively, in the high density and low density region. The pore sizes of the foam via ET are 60 μm and 80 μm, respectively, in the high density and low density region (Figure 5B).

In the scaffold preparation process via microfluidic emulsion templating, droplet generation is an important and first step [41,86]. In recent years, there have been many reports on control of droplet generation by microfluidic technology [40,41,53,87]. Microfluidic droplets can be generated in either passive or active manners [40]. In the passive manner, a microfluidic two-phase flow is usually controlled by syringe pumps without additional energy input. By changing the size and geometry of the microfluidic flow focusing junction, the flow rates of the two-phase, type and concentration of surfactant, it is possible to tune the frequency of generation of droplets, their diameter, and the volume fraction of the dispersed phase of the emulsion [40,44]. For instance, a capillary microfluidic device was used for fabrication of a gelatin methacryloyl foam (Figure 5D), where the orifice size was approximately 80-150 μm. The flow rate of the dispersed phase was set at 0.1-0.5 mL/h and that of continuous phase was set at 1-5 mL/h. The results demonstrated that the size of the droplets increased with an increase in the dispersed flow rates but decreased as the continuous flow rates increased [51]. Compared with passive manner, active methods need to add external forces, including electrical, magnetic and centrifugal fields, to modulate droplet formation [88]. Active droplet generation allows more flexible control of droplet size and production rate, which provides a strong basis for the preparation of well-ordered or tailor-made scaffolds [89]. 

The emulsion templating technique combined with microfluidics represents a perfect basis for the design of well-ordered and tailored scaffolds. Nevertheless, one of its challenges and the limitations is how to remove the template, which makes this a high-cost procedure for large-scale production. It takes time to remove the solvent, and there is the possibility of incomplete removal. Meanwhile, the solvents used in removal of the template and residual template have an impact on the biocompatibility of scaffolds. Therefore, choosing the appropriate emulsion composition and using biocompatible solvent to remove template may be one way to solve this issue. The third type of the templates, i.e., hard templates, has been proposed to prepare scaffolds with well-controlled structure recently. This mainly concerns sacrificial CaCO_3_ templates, which can be removed at truly mild conditions [90,91,92]. Paulraj et al. [93] prepared polymeric 3D scaffolds through that the crystals of CaCO_3_ were packed and coated with polymer layer-by-layer in the microfluidic chamber. Apart from that, porous scaffolds for TE often require bioactive nanoparticles induce tissue regeneration. However, the obtained-scaffolds usually contain a single polymer, lacking of biological activity [94,95,96]. One approach to this issue is Pickering emulsion templating, which stabilized by the solid particles [97,98]. Solid particles are non-toxic, provide support features (such as magnetism, electric conductivity, thermal conductivity, etc.) and also enhance the mechanical properties of porous scaffolds. Thus, well-ordered porous composite scaffolds may be fabricated by combining Pickering templating with microfluidics, and that will have important implications for studying the effects of nanoparticles in scaffolds on cellular behaviors.

## 4. Microfluidic Scaffolds and Biomedical Applications

**Figure 6:**
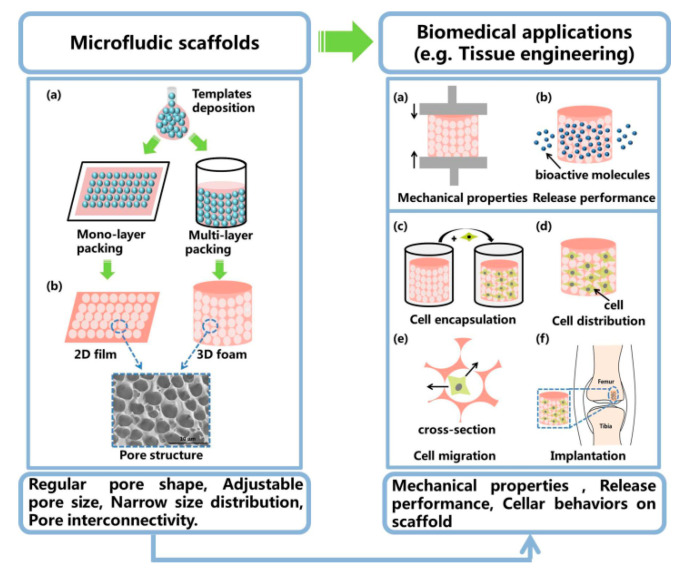
Schematic diagram: microfluidic scaffolds and their biomedical applications, especially in TE.

As mentioned above, well-ordered or tailor-made porous scaffolds were obtained through microfluidic template method. For the sake of simplicity, we call the foamed scaffold as microfluidic scaffold in this review. Microfluidic templates assembled into either monolayer or multilayer packings and formed into 2D film-like scaffolds and 3D foam-like scaffolds (Figure 6) [30]. These scaffolds have special physical properties and unique advantages for biomedical applications, especially in tissue engineering, drug delivery system and cellular responses in different surface morphologies.

### 4.1. 2D Film-Like Scaffolds

The microfluidic template method offers a powerful tool for precisely engineering microstructured material networks. After monodisperse microfluidic template monolayer packing, the liquid phase solidifies and compresses microdroplets into different shapes to template microstructures, then, polymer films composed of microstructures are obtained by removing the deformed droplet templates. These membranes have ordered porous structure, including tunable pore sizes, regular pore shape and narrow the pore size distribution, for example, honeycomb-patterned films. The resulting well-ordered films have elicited much interest in many areas such as microarrays and as scaffolds for TE [48]. 

**Figure 7:**
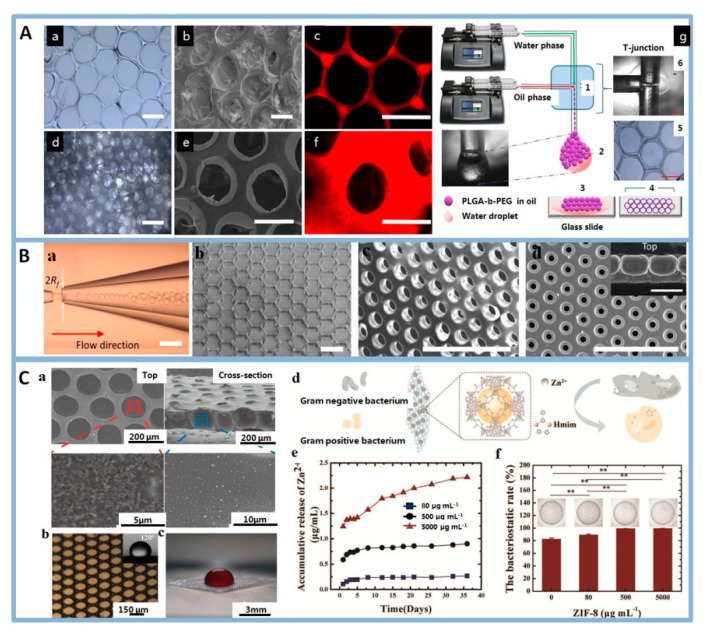
(**A**): Optical micrographs (a,d), SEM (b,e), and fluorescent microscopes (c,f) of honeycomb-like structures, (g) schematic preparation process of scaffolds with T-junction microfluidic device, scale bars indicate 100 μm (a,b,d,e), 200 μm (c), and 400 μm (f). Reprinted from [28] with permission. Copyright 2018 American Chemical Society. (**B**): (a) micrographs of microfluidic devices used for the generation of droplets, monolayer film of (b) the cage, (c) through-pore, and (d) dead-end pore structure of the film. Reprinted from [30] with permission. Copyright 2018 Wiley. (**C**): (a) SEM images showing the porous structure from a top view and a cross-sectional view, and ZIF-8 inside the membranes, (b) microscopic optical image of the membrane with uniform pores, (c) optical image showing a drop of blood on the porous membrane, (d) schematic diagrams for the antimicrobial mechanism of ZIF-8 hydrogel membranes, (e) The zinc-ions release of the ZIF-8-laden membranes with different ZIF-8 concentrations during 36 days, (f) The statistical graph of the bacteriostatic rate of the membranes at different ZIF-8-laden concentrations. Reprinted from [84] with permission. Copyright 2020 Wiley.

In recent years, well-ordered alginate films [27], PLGA-*b*-PEG films [28] and PVA films [29,30] were manufactured by microfluidic template. Edirisinghe et al. used microfluidic bubbles [27] and microfluidic droplets [28] to produce highly oriented porous films with surface-embedded nanparticles by microfluidic device in a controlled way (Figure 7A). These structures were helpful in applications that require particular surface properties [99]. For example, Zhu et al. [29,30] designed and produced robust omniphobic membranes with well-defined interconnected micro-cavity structures by evaporation-induced self-assembly of microdroplets (Figure 7B).

Highly ordered porous polymer structures have great potential in biomedical application. In TE, the pore architectures of the porous film affected the extent of cellar behaviors, including adhesion, proliferation, migration, differentiation [25,100,101]. For example, the morphology and functionality of cardiac myocytes, neural progenitor cells, and endothelial cells were manipulated by altering the size and shape of the micropores of the honeycomb films [102]. Eniwumide et al. [103] studied the behaviors of chondrocytes on honeycomb-patterned films. The results demonstrated that micro-patterned surfaces can enhance proliferation of chondrocytes compared with non-patterned surfaces and maintain the cells phenotype over a prolonged culture period. Besides, well-ordered porous films widely applied in wound healing and drug delivery system. Yao et al. [84] prepared omniphobic well-ordered porous hydrogel based on droplet microfluidics. The hydrogel could inhibit bacteria invasion and release zinc-ions in a controlled manner (Figure 7C).

### 4.2. 3D Foam-Like Scaffolds

Microfluidic templating technique offers a new method for finely controlling pore size and connectivity with acceptable reproducibility. Well-ordered porous 3D foams are obtained by microfluidic template multilayer-packing (Figure 6). Microfluidic foams usually have uniform pore size and narrow pore size distribution [60]. 

**Figure 8:**
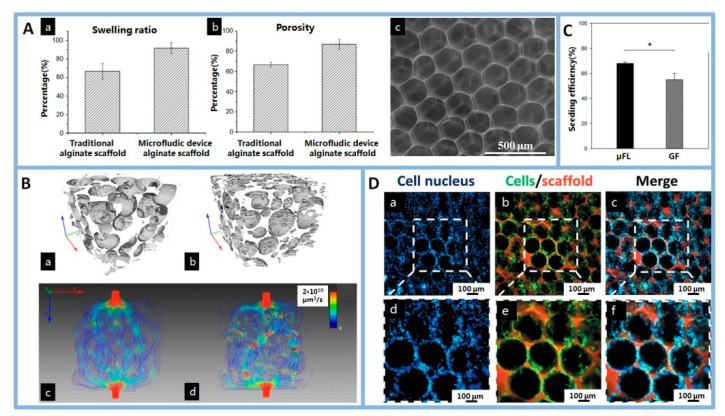
(**A**): (a) swelling ratio and (b) the average porosity of microfluidic alginate scaffold and the traditional alginate scaffold, (c) SEM of microfluidic alginate scaffold. Reprinted from [104] with permission. Copyright 2011 Elsevier. (**B**): 3D reconstructions of μFL (a) and GF scaffolds (b) from microcomputed tomography (μCT) data, volumetric fluid flow through μFL (c) and GF (d) scaffolds as calculated by CFD simulations, μFL: microfluidic foaming scaffold, GF: gas-in-liquid foam templating scaffold. Reprinted from [67] with permission. Copyright 2016 Elsevier. (**C**): cell seeding efficiency values at 24h for μFL and GF scaffolds. Reprinted from [67] with permission. Copyright 2016 Elsevier. (**D**): After culture 7 days, cells distribution on the wall of the scaffold, most cells were stained with green fluorescence and the merged images revealed that cells had a good survival rate. Reprinted from [64] with permission. Copyright 2014 Wily.

Wang et al. [104] prepared an alginate scaffold by using a microfluidic device. Compared with traditional scaffolds, microfluidic scaffolds have highly organized structures resembling a honeycomb framework, which exhibited high swelling ratio and porosity (Figure 8A). Importantly, their porosity, the pore size and interconnects between the pores were precisely adjusted.

In TE scaffolds, the pore structure of the 3D foams plays a key role in the mechanical properties, related biological absorbability as well as the ability to release biomolecules at an ideal rate. TE scaffolds should match those of the host tissue and mechanically strong enough to remain intact until new tissue is regenerated [105,106,107,108]. Microfluidic foams have regularity of pore size and geometries, results in homogeneous cross-linking throughout the polymeric structures, and improve the mechanical properties of scaffolds [85,109]. In addition, microfluidic scaffolds with uniform pore size degrade more evenly, and the overall performance of scaffolds changes more evenly [110]. Besides, growth factor plays a significant role in promoting the formation of new tissue [111,112]. Previous studies showed that the pore structure of the scaffolds plays an important role in controlling drug-loading efficiency and the release behaviors [113]. The pore size of microfluidic scaffolds can be adjusted and precisely regulated, therefore, the drug loading efficiency and release behavior can also be precisely regulated in the process by adjusting the pore structure.

Porous scaffolds provide a spatial conformation and orientation to cells, playing a pivotal role in cellular behavior. A regular pore shape and ordered pore arrays are the basis for achieving uniform cell distribution in the implanted scaffolds [114]. For example, in bone TE, the ordered geometry of hyaluronic acid (HA) foams cause collagen to self-assemble in the pores to form dense lamellar bone. In contrast, HA/collagen composites with disordered pore structures initiate collagen deposition in the nematic phase, resulting in the formation of woven ectopic bone [115]. Costantini et al. [66,67] prepared highly ordered polymer scaffolds by microfluidic foaming. Simulation of local flow velocity inside the scaffolds provided that microfluidic scaffolds were around one order of magnitude more permeable than traditional scaffolds (Figure 8B). Cell seeding revealed that cell proliferation inside microfluidic scaffolds was quantitatively higher than in traditional scaffolds (Figure 8C). Meanwhile, the size of the internal interconnections and their direction determined the flow direction, path and speed, influencing the supply of nutrients and oxygen and the disposal of metabolic wastes throughout the whole scaffolds, so the size of pores and interconnects were extremely important not only during cell seeding but also cell migration and differentiation within the scaffolds. Wang et al. [64,104] produced a highly organized honeycomb-like scaffold using microfluidic device. The results of chondrocytes culture on scaffolds indicated that this scaffold presented excellent performance in cartilage tissue engineering (Figure 8D).

## 5. Conclusions

The pore structure of scaffolds has a great influence on their mechanical properties, degradation rate, permeability properties and cellular behaviors on them. Both traditional and advanced methods are trying to prepare scaffolds to meet various requirements, but the effect is not obvious, such as non-uniform pore sizes, poor repeatability. This review summarized the latest research in the preparation of well-ordered porous scaffolds via microfluidic technology. By using microfluidic technology, it is possible to precisely adjust the size and size distribution of the template, hence, providing an access to prepare various foams with monodisperse, polydisperse and graded structures. These foams are widely used in tissue engineering and drug delivery system. Microfluidic technology offered a platform for the preparation of tailor-made porous scaffolds, which is of great benefit to the further study of the mechanism of tissue formation such as the role of cells and scaffolds in TE.

Nevertheless, one of the major challenges is the rate of production, which is a common problem of microfluidic technology. Parallelization of identical microfluidic channels to scale up the production has been successful attempted in emulsions, but the parallelization of controlled bubble generation still remains a challenge. Additionally, monodisperse and polydisperse foams are mostly discussed, while scaffolds with bi-modal size distributions are more interesting in TE. This has not been realized, because bimodal foams do not self-order spontaneously and an external force is required to drive the initially disordered foam into the crystal structure. Last but not least, the biocompatibility and bioactivity of microfluidic foams still need to be improved and further studied. The combination of microfluidics with hard templates or Pickering emulsions maybe a solution to this issue. Once these problems are addressed, the superiority of microfluidics will be fully displayed and they may play a much more important role in preparing tissue engineering scaffolds.

## Figures and Tables

**Table 1:** The advantages and limitations of conventional methods for engineering scaffolds.

Methods	Pore Structures	Advantages and Limitations	Refs.
Freeze-drying technique	The pore sizes and porosity depend on the ratio of water to polymer solution and the viscosity of emulsions. Pore size < 100 μm, porosity is low.	Advantages: the elimination of several rinsing steps. Limitations: Small and disordered pores and residual solvent is dangerous for cells.	[13,14]
Gas foaming	The pore sizes and porosity are dependent on the pressure of carbon dioxide gas. Pore size > 100 μm and porosity > 93%.	Advantages: the elimination of the use of harsh chemical solvents. Limitations: it is difficult to ensure pore connectivity and control of the pore sizes.	[15,16]
Solvent casting/ particulate leaching	The pore size and geometry are determined by size and geometry of porogen, And the porosity depends on the size of the porogen and the ratio of polymer to porogen. Pore size: 50–500 μm and porosity > 80%.	Advantages: the use of small amounts of polymer. Limitations: the interpore openings and pore shape is not controllable, and takes a long time to remove the porogen, residual porogen is hazardous for cells.	[17,18,19]
Emulsion template	The pore size: 100 nm–2 mm and porosities (60–97%). The size distribution is highly dispersed.	Advantages: practical and inexpensive, tailoring the properties of materials flexibly. Limitations: the surfactant is difficult to remove and has certain toxicity to cells.	[20,21]
Electrospinning	The microstructure: nanofibrous networks and the size range from nanometer to micrometer.	Advantages: high porosity and high surface-to-volume. Limitations: the electrospun membrane is 2D structure.	[22,23,24]

**Table 2:** The advantages and limitations of existing methods for preparing well-ordered porous scaffolds.

Methods	Pore Structures	Advantages and Limitations	Refs.
Breath figure	The pore size is largely dependent on polymer property, temperature and humidity. Pore shape is regular and pore size is uniform. Pore size: 0.2–20 μm.	Advantages: simple and cheap. Limitations: the temperature and humidity must be strictly controlled and volatile solvents should be used. And the materials are limited to amphiphilic copolymer.	[25,26]
Microspheres cubic close-packed	The pore structure is largely dependent on microspheres template. And the obtained scaffolds have high porosity, pore size uniformity and good connectivity. Pore size: 0.05–500 μm.	Advantages: the ideal structure of scaffold. Limitations: multi-step operation and organic solvent are needed; solvent residue may be a biological toxicity affect the adhesion of cell growth.	[31,32]
3D printing	The pore sizes, pore morphology and porosity are controlled by design and process.	Advantages: the fabrication of more complex shapes and controlled internal structures. Limitations: High precision equipment is expensive and applicable materials are limited.	[33,34,35,36,37]
